# *E2F7* serves as a potential prognostic biomarker for lung adenocarcinoma

**DOI:** 10.1097/MD.0000000000034342

**Published:** 2024-01-19

**Authors:** Shengcheng Lin, Xiangyang Yu, Haojie Yan, Yafei Xu, Kai Ma, Xiaoliang Wang, Yeqing Liu, Ahuan Xie, Zhentao Yu

**Affiliations:** a Department of Thoracic Surgery, National Cancer Center/National Clinical Research Center for Cancer/Cancer Hospital and Shenzhen Hospital, Chinese Academy of Medical Sciences and Peking Union Medical College, Shenzhen, China; b Translational Medicine Collaborative Innovation Center, Shenzhen People’s Hospital (The Second Clinical Medical College, Jinan University; The First Affifiliated Hospital, Southern University of Science and Technology), Shenzhen, China; c Basic Medicine Postdoctoral Research Station, Jinan University, Guangzhou, China; d Department of Anesthesiology, Shunde Hospital of Southern Medical University (The First People’s Hospital of Shunde Foshan), Foshan, China; e Department of Pathology, National Cancer Center/National Clinical Research Center for Cancer/Cancer Hospital and Shenzhen Hospital, Chinese Academy of Medical Sciences and Peking Union Medical College, Shenzhen, China.

**Keywords:** bioinformatic analysis, E2F transcription factor (*E2F7*), lung adenocarcinoma, prognostic biomarker

## Abstract

E2F transcription factors (E2Fs) are a family of transcription factors critical regulators of the cell cycle, apoptosis, and differentiation, thus influencing tumorigenesis. However, the specific roles of E2Fs in lung adenocarcinoma (LUAD) remain unclear. Data from The Cancer Genome Atlas (TCGA) were used. R version. 4.0.3 and multiple databases (TIMER, cBioportal, gene expression profile interaction analysis [GEPIA], LinkedOmics, and CancerSEA) were utilized to investigate mRNA expression, mutational analysis, prognosis, clinical correlations, co-expressed gene, pathway and network, and single-cell analyses. Immunohistochemistry (IHC) confirmed that E2F transcription factor 7 (*E2F7*) correlated with LUAD. Among the E2Fs, *E2F7* was identified by constructing a prognostic model most significantly associated with overall survival (OS) in LUAD patients. The univariate and multivariate Cox regression analyses showed that *E2F7*, p-T stage, and p-TNM stage were closely related to OS and progression-free survival (PFS) (*P* < .05) in LUAD. E2F 7/8 were also identified as significantly associated with tumor stage in the GEPIA database. Compared with paracancerous tissues, *E2F7* was up-regulated in LUAD by IHC, and *E2F7* might be positively correlated with larger tumors and higher TNM stages. *E2F7* may primarily regulate DNA repair, damage, and cell cycle processes and thus affect LUAD tumorigenesis, invasion, and metastasis by LinkedOmics and CancerSEA. *E2F7* serves as a potential prognostic biomarker for LUAD.

## 1. Introduction

Lung adenocarcinoma (LUAD), the most frequent histological subtype of lung cancer, accounts for ~50% of all cases of lung cancer.^[1]^ With developments in diagnosis, surgery, chemotherapy, radiotherapy, molecular therapy, and immunotherapy, the mortality of LUAD has greatly declined.^[2]^ However, the 5-year survival rate is still <15% due to the propensity of many LUAD patients to be diagnosed with metastatic disease.^[2]^ The main challenge in managing LUAD patients is the inability to effectively distinguish between inert and invasive tumors. Thus, there is an urgent need to identify potential prognostic biomarkers to provide value for individualized treatment and effective improvements in the prognosis of LUAD.

E2F transcription factors (E2Fs) are a large family of transcription factors that serve as key regulators of eukaryotic cell cycles, apoptosis, and differentiation.^[3]^ In general, E2F family genes encode 3 activators (*E2F1, E2F2*, and *E2F3A*) and 6 repressors (sub-classified into canonical (*E2F3B, E2F4, E2F5*, and *E2F6*) and atypical (E2F transcription factor 7 [*E2F7*] and *E2F8*) repressors.^[4]^ All E2F proteins harbor evolutionarily conserved DNA-binding domains that bind to the 5’-TTT[CG][CG]CGC-3’ sequence of the target promoter and regulate its expression.^[4]^ Owing to interactions with its major binding partner and crucial regulator, retinoblastoma, the overexpression of cyclin-dependent kinases, or inhibitor inactivation, E2Fs are widely observed in virtually all human cancers,^[5]^ including lung,^[6]^ breast,^[7]^ bladder,^[8]^ colorectal,^[9]^ and prostate^[10]^ cancer.

As essential components of biological and biomedical research, RNA and DNA research has been revolutionized by the development of microarray technology.^[11]^ In this study, we collected the expression profiles of the E2F genes from the Tumor Immune Estimation Resource (TIMER2.0) and the Cancer Genome Atlas Data (TCGA) database. Then we acquired the E2F gene expression and genomic alteration analysis from the cBioportal for Cancer Genomics. A prognostic model was also established using univariate and multivariate Cox regression analyses and least absolute shrinkage and selection operator (LASSO) to determine which E2Fs were significantly associated with OS and progression-free survival (PFS). Subsequently, we investigated the Gene Expression Profile Interaction Analysis (GEPIA) database and determined the association between E2F transcript levels and clinicopathological parameters in LUAD patients and validated by immunohistochemistry (IHC). Finally, we performed co-expressed gene, pathway and network, and single-cell analyses to further examine the effects and predictive value of elevated prognostic gene levels in LUAD patients using the LinkedOmics and CancerSEA databases. Taken together, in the present data, we examined the expression, clinical relevance, and molecular mechanisms of *E2F7* in LUAD.

## 2. Methods

### 2.1. Ethics statement

The study was approved by the Cancer Hospital and Shenzhen Hospital, Chinese Academy of Medical Sciences and by the Peking Union Medical College Institutional Review Board of Clinical Research (proposal no.: KYKT2021-2-1). It was conducted in accordance with the Declaration of Helsinki. All datasets are available from the published literature.

### 2.2. TIMER database

TIMER2.0^[12]^ (http://timer.cistrome.org/) an open-access cancer microarray database for web-based data mining, was used to analyze the E2F expression profiles across diverse cancer types.

### 2.3. cBioportal and TCGA database

The cBioportal^[13]^ (http://cbioportal.org) a publicly accessible online platform, was used to analyze the somatic mutations and co-expression of E2Fs in LUAD datasets from the TCGA database, a landmark cancer genomics program,^[14]^ with 507 pathologically reported cases.

### 2.4. Statistical analysis

In January of 2020, the level-3 RNA sequencing data and corresponding clinicopathological parameters from 504 LUAD patients were acquired from the TCGA dataset, according to the relevant guidelines and policies for data acquisition and application. The R v. 4.0.3 (codename: “Bunny-Wunnies Freak Out”), package “*ggplot2*” and “*pheatmap*” was applied to perform Wilcoxon rank test to compare the expression levels of E2Fs between LUAD and normal controls. The package “*glmnet*”^[15]^ was employed in the regression algorithm of the LASSO for feature selection, using 10-fold cross-validation. The “*survminer*” package^[16]^ was used to acquire the median cutoff value and to calculate the risk score of LUAD patients, who were sub-classified by the median risk score, including high- and low-risk groups. Survival outcomes in the high- and low-risk groups were compared using Kaplan–Meier (KM) survival analysis and log-rank tests. Hazard ratios with 95% confidence intervals and *P* value < .05 were generated using log-rank and Cox proportional hazards tests to compare KM curves between the 2 groups.^[16]^ We also performed a “*timeROC v. 0.4*”^[17]^ analysis to compare the predictive accuracy of the risk score and E2Fs. Univariate and multivariate Cox regression analyses were conducted to identify terms that could be used for nomogram construction. Forest plots showing *P* value, HRs, and 95% CIs for various variables were implemented with the “forestplot” package in R. For all analyses, *P* < .05 was considered statistically significant.

### 2.5. GEPIA

GEPIA (http://gepia.cancer-pku.cn/) a publicly accessible interactive web server for analyzing RNA sequencing data, was used to determine the correlations between E2F transcript levels and clinicopathological parameters in LUAD.^[18]^

### 2.6. Immunohistochemistry

The 3-μm LUAD and paracancerous tissues sections were incubated with a commercial rabbit polyclonal antibody against *E2F7* (Aviva Systems Biology, San Diego, CA) at a dilution of 1:100 at 4°C overnight. Sections were then placed with horseradish peroxidase antibody (1:500 dilution; MXB, Fuzhou, Fujian, China) for 30 minutes at room temperature, then covered with DAB (MXB, Fuzhou, Fujian, China) and slides were mounted, observed and photographed with a confocal microscope (Leica, Germany).

### 2.7. LinkedOmics

LinkedOmics (http://www.linkedomics.org/login.php), an open-access online portal that includes multi omics cancer data, was used to obtain the *E2F7* co-expression evaluated in LUAD via Pearson correlation coefficient and shown in volcano and heat maps.^[19]^ The LinkInterpreter module was employed to analyze gene ontology (GO),^[20]^ including molecular functions, cellular component, and biological processes, and the Gene Set Enrichment Analysis tool was applied to Kyoto Encyclopedia of Genes and Genomes pathways.^[21]^ After 500 simulations, the rank criterion was set as *P* < .05.

### 2.8. CancerSEA

CancerSEA (http://biocc.hrbmu.edu.cn/CancerSEA/), a functional state landscape of a single cell in cancer, was used to comprehensively observe the potential roles of *E2F7* in LUAD at the single-cell level^[22]^ and to profile the 14 functional states associated with LUAD.

## 3. Results

### 3.1. The mRNA expression and mutational analysis of E2Fs in LUAD

**Figure 1.**
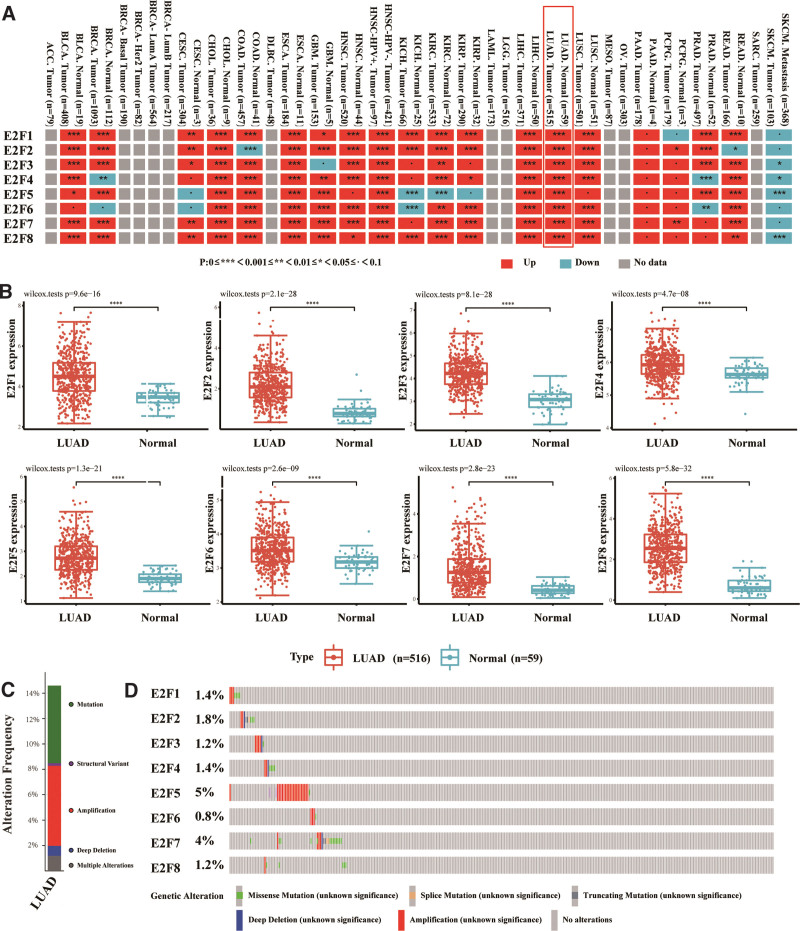
The expression and genetic alterations in the E2Fs in LUAD. (A) The expression profiles of the E2Fs genes in the TCGA dataset (TIMER). (B) The expression profiles of E2Fs in the 516 LUAD and 59 normal samples in the TCGA-LUAD dataset. (C) Synopsis of alterations in differentially expressed E2F genes in LUAD. (D) E2F gene expression and genomic alteration analysis in LUAD (cBioPortal). E2Fs = E2F transcription factors, LUAD = lung adenocarcinoma, TCGA = The Cancer Genome Atlas.

In the TIMER2.0 database, the E2F genes were frequently dysregulated in cancers with a cancer-specific pattern. For example, *E2F7* was upregulated in bladder urothelial carcinoma, breast cancer, cholangiocarcinoma, colon adenocarcinoma, esophageal carcinoma, glioblastoma, head and neck squamous cell carcinoma, kidney renal clear cell carcinoma, kidney renal papillary cell carcinoma, liver hepatocellular carcinoma, LUAD, lung squamous cell carcinoma, rectum adenocarcinoma (*P* < .001). It was also upregulated in cervical squamous cell carcinoma and endocervical adenocarcinoma and pheochromocytomas and paragangliomas (*P* < .01), while no statistical difference was found in suspicious cell carcinoma (KICH), pancreatic adenocarcinoma, prostate adenocarcinoma, and skin melanoma (*P* < .1), and no data were available for other tumors (Fig. 1A). In TCGA-LUAD dataset, E2F genes were found to be significantly upregulated in LUAD compared with normal controls (Fig. 1B and Supplemental Digital Content [Table S1, http://links.lww.com/MD/J286, and Table S2, http://links.lww.com/MD/J287]). Using the cBioPortal online tool,^[13]^ we analyzed alterations in and the co-expression of E2Fs in LUAD based on Cancer Genome Atlas and Pan-Cancer Atlas data (14). Among the 507 LUAD patients, E2Fs were mutated in 74 cases (15%) (Fig. 1C). The genomic mutation rate of E2F5 was the highest within the E2F family, accounting for 5%, followed by *E2F7* (4%), *E2F2* (1.8%), *E2F1/4* (1.4%), and *E2F3/8* (1.2%), while *E2F6* had the lowest mutation rate (0.8%) (Fig. 1D).

### 3.2. Construction of a prognostic model for LUAD patients based on E2Fs

**Figure 2.**
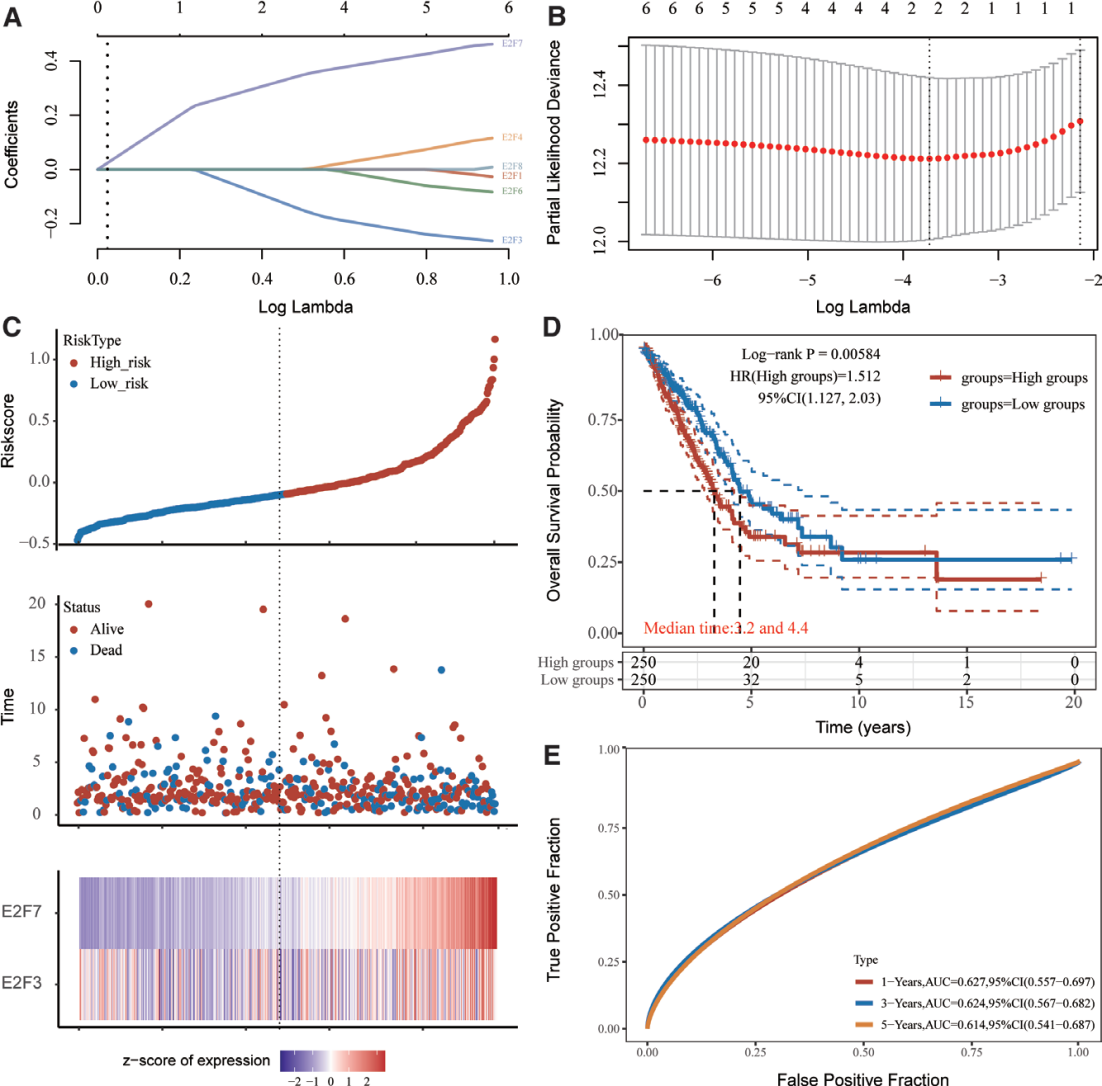
Prognostic analysis of E2Fs in LUAD patients. (A) LASSO coefficient distribution of E2Fs. (B) LASSO regression with tenfold cross-validation using the minimum λ value obtained for 2 prognostic genes. (C, upper) Distribution of risk scores in LUAD patients, who were divided into high-risk (red dots) and low-risk (blue dots) groups according to their median (dashed line) OS. (C, middle) Distribution of survival status in LUAD patients. Red dots indicate death and green dots indicate survival. (C, lower) Heat map describing E2F expression patterns between high- and low-risk groups. (D) KM survival curves indicating the overall difference in survival between the high-risk and low-risk groups. (E) Time-dependent ROC analysis of 2-gene prognostic features. E2Fs = E2F transcription factors, KM = Kaplan–Meier, LUAD = lung adenocarcinoma, OS = overall survival, ROC = receiver operating characteristics.

In January of 2020, the level-3 RNA sequencing data and corresponding clinicopathological parameters from 504 patients with LUAD were acquired from the TCGA dataset. LASSO regression was performed to determine the optimum lambda value from the minimum partial likelihood deviation (λmin = 0.025), which was correlated with 2 genes in differentially expressed genes significantly related to overall survival (OS), to further identify the E2Fs that were significantly correlated with the clinical outcomes of LUAD patients (Fig. 2A and B and, Supplemental Digital Content [Table S3, http://links.lww.com/MD/J288]). We determined that *E2F7* and *E2F3* were optimal for the model via best subset regression analysis because they had the lowest Akaike information criteria. The risk score was then calculated and the median cutoff value for LUAD patients was acquired. The LUAD patients were sub-classified according to median risk score, including high-risk (n = 252) and low-risk (n = 252) groups (Fig. 2C and Supplemental Digital Content [Table S3, http://links.lww.com/MD/J288]). The number of LUAD patients who died steadily increased with the risk score (Fig. 2C), and the transcript levels of the 2 genes were significantly different between the 2 groups (Fig. 2C and, Supplemental Digital Content [Table S3, http://links.lww.com/MD/J288]). According to the results of KM analysis, the high-risk group showed a shorter OS time than the low-risk group (*P* = .006, Fig. 2D and, Supplemental Digital Content [Table S3, http://links.lww.com/MD/J288]). The sensitivity and specificity of the risk scores were also evaluated using timeROC analysis, and the areas under the receiver operating characteristics curves were 0.627, 0.624, and 0.614 for 1-, 3-, and 5-year OS, respectively (Fig. 2E and Supplemental Digital Content [Table S3, http://links.lww.com/MD/J288]).

### 3.3. The prognostic role of E2Fs genes

**Figure 3.**
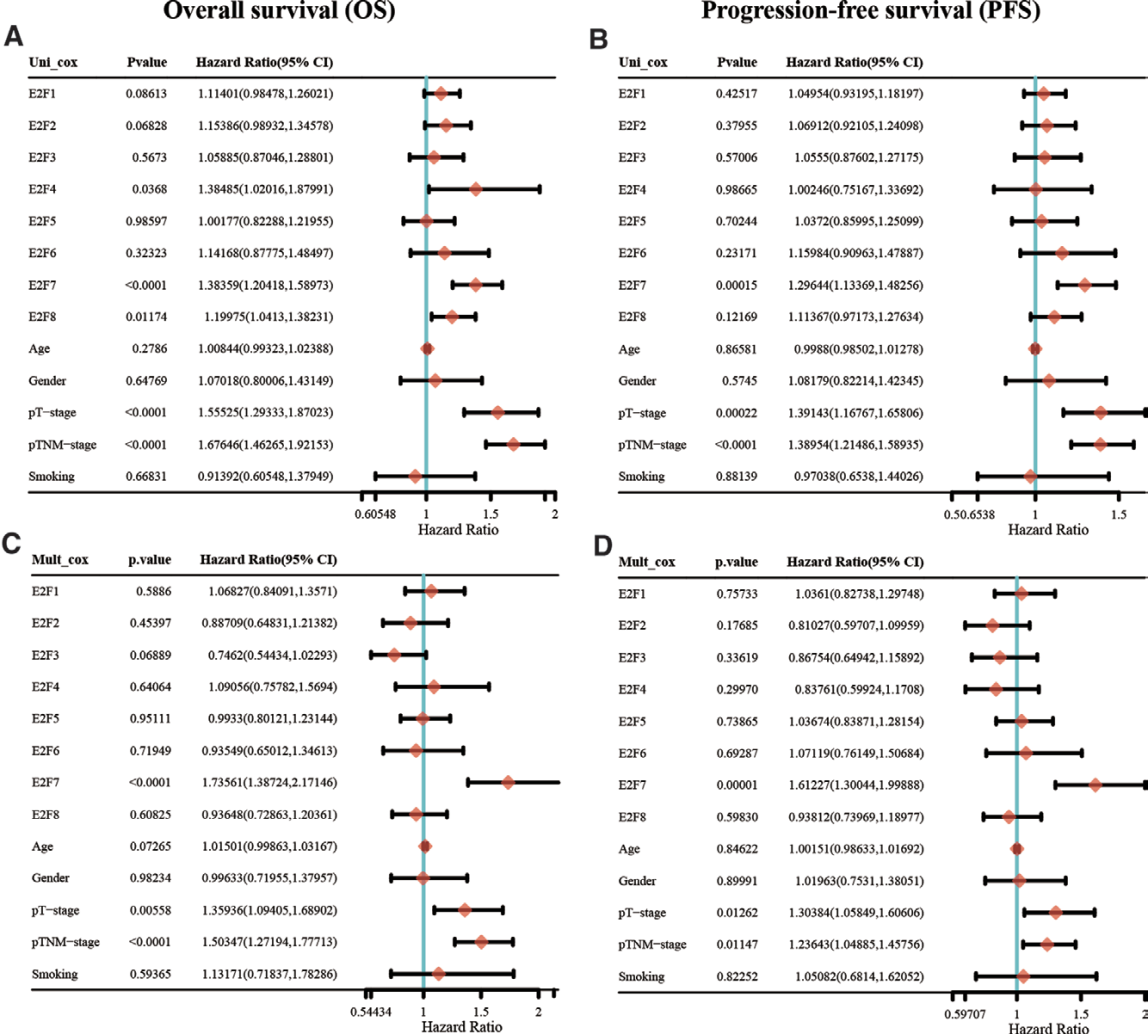
Correlation of prognostic E2Fs and clinical characteristics. (A and C) Univariate and multivariate COX regression analyses of overall survival (OS) using the Cox proportional hazard regression mode. (B and D) Univariate and multivariate COX regression analyses of progression-free survival (PFS) using the Cox proportional hazard regression mode. E2Fs = E2F transcription factors.

For further analysis of relevant clinicopathological factors (E2F genes, patient age, patient gender, p-T stage, p-TNM stage and smoking) that may affect OS and PFS, we performed univariate and multivariate Cox regression analyses in the TCGA LUAD cohort. *E2F7*, p-T stage and p-TNM stage were closely related to OS (*P* < .05) and PFS (*P* < .05) in LUAD (Fig. 3A–D and, Supplemental Digital Content [Table S4, http://links.lww.com/MD/J289, and Table S5, http://links.lww.com/MD/J290]).

### 3.4. Correlations between E2F expression and LUAD patient clinicopathology

**Figure 4.**
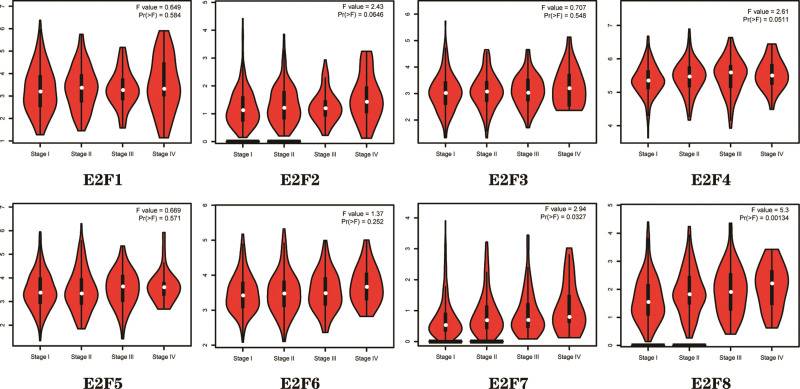
Correlation between E2F transcript levels and tumor stage in LUAD Patients (GEPIA). GEPIA = Gene Expression Profile Interaction Analysis, LUAD = lung adenocarcinoma.

Using the GEPIA dataset,^[18]^ we compared the E2F expression in pathological stages. Correlations between E2F expression and tumor stage in LUAD patients were also analyzed and we found that those of *E2F7*/8 were significantly associated with tumor stage (*P* < .05), while those of *E2F1/2/3/4/5/6* were not (*P* > .05) (Fig. 4).

### 3.5. *E2F7* Expression detected by IHC in LUAD patients

**Figure 5.**
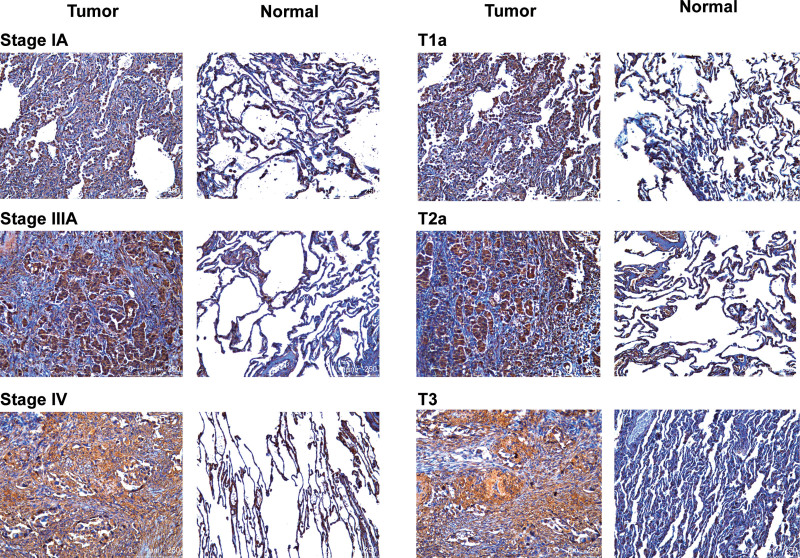
*E2F7* transcript levels in LUAD (IHC). *E2F7* = E2F transcription factor 7, IHC = immunohistochemistry, LUAD = lung adenocarcinoma.

To verify whether *E2F7* is significantly associated with the occurrence and development of LUAD, we analyzed *E2F7* protein expression in LUAD and paracancerous tissues using IHC. Compared with paracancerous tissues, *E2F7* was up-regulated in LUAD, and *E2F7* was positively correlated with larger tumors and higher TNM stage (Fig. 5).

### 3.6. Gene co-expression with *E2F7* in LUAD

**Figure 6.**
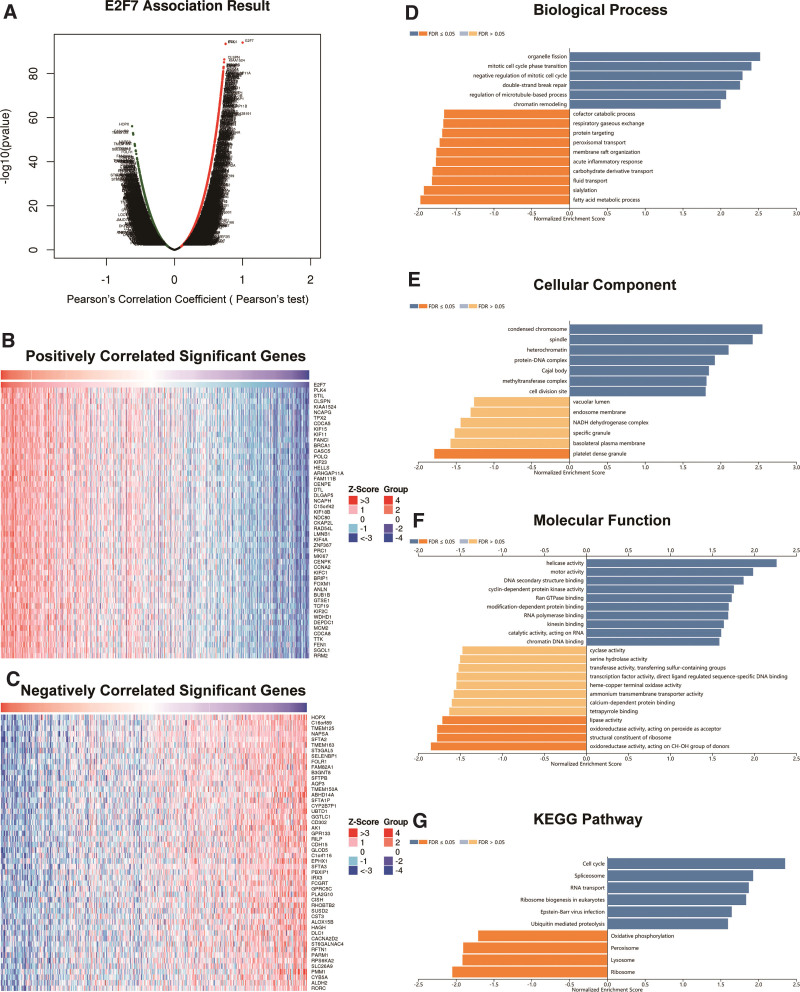
Co-expression of genes with *E2F7* in LUAD (LinkedOmics). (A) Volcano plot showing that *E2F7* is highly relevant in the LUAD cohort identified by Pearson correlation analyses; dark red represents positively associated genes, and dark green represents negatively associated genes. (B and C) Heat maps showing the top 50 significantly positively and negatively correlated genes with *E2F7* in LUAD. Genes co-expressed with *E2F7* in LUAD were significantly enriched for GO annotation and KEGG pathways using GSEA. (D) BP, (E) CG, (F) MF, and (G) KEGG pathway analyses. The *x*-axis indicates the normalized enrichment fraction, and the *y*-axis indicates the GO term. BPs = biological processes, *E2F7* = E2F transcription factor 7, GO = gene ontology, GSEA = Gene Set Enrichment Analysis, KEGG = Kyoto encyclopedia of genes and genomes, LUAD = lung adenocarcinoma.

We used the functional module of LinkedOmics^[19]^ to detect the patterns of gene co-expression with *E2F7* in the TCGA LUAD cohort (n = 515) to gain insight into the biological significance of *E2F7* in LUAD cells. Volcano plots revealed that 5143 (dark red dots) and 4487 (dark green dots) genes were significantly positively and negatively correlated with *E2F7*, respectively (FDR < 0.01), and heat maps showed the top 50 significantly correlated genes (Fig. 6A–C and, Supplemental Digital Content [Table S6, http://links.lww.com/MD/J291]). The full description of the co-expressed genes is provided in Table S6, http://links.lww.com/MD/J291. The expression of *E2F7* was positively correlated with those of *PLK4 (P* = 3.251e-94), *STIL (P* = 4.254e-94), and *CLSPN (P* = 7.307e-01), etc. Notably, the top 50 significantly positive genes (high hazard ratio, HR) were highly likely to be high-risk in LUAD (*P* < .05). In contrast, the top 50 significantly negative genes (low HR) were highly likely to be low-risk (*P* < .05) (Fig. 6C). GO analysis of gene set enrichment analysis on LinkedOmics revealed that the differentially expressed genes related to *E2F7* were mainly located in the negative regulation of the mitotic cell cycle (GO: 0045930), followed by the mitotic cell cycle phase transition (GO: 0044772), and cell division site (GO: 0032153), which are primarily involved in the cell cycle (Fig. 6D–F and, Supplemental Digital Content [Table S7, http://links.lww.com/MD/J292]). Additionally, Kyoto Encyclopedia of Genes and Genomes pathway analysis showed enrichment in the cell cycle (hsa04110), spliceosome (hsa03040), and RNA transport (hsa03013) (Fig. 6G and, Supplemental Digital Content [Table S8, http://links.lww.com/MD/J293]).

### 3.7. Functions of *E2F7* in LUAD cells

**Figure 7.**
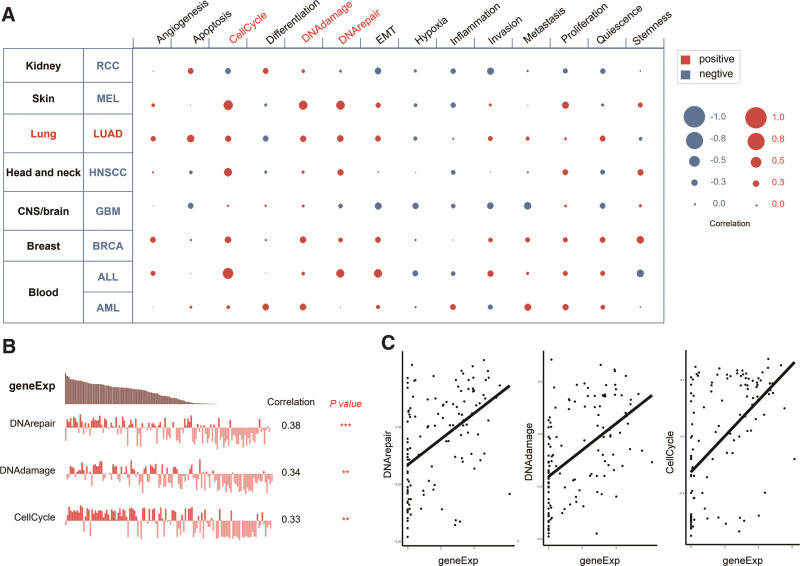
Functions of *E2F7* in LUAD cells. (A) Single-cell analysis demonstrated that *E2F7* is mainly involved in the regulation of DNA repair and damage and the cell cycle in LUAD. (B and C) Data from Kim et al (42) (cell no. = 126) indicated that *E2F7* transcript levels are positively correlated in LUAD by regulating DNA damage and repair and the cell cycle. *E2F7* = E2F transcription factor 7, LUAD = lung adenocarcinoma.

Using CancerSEA, the functions of *E2F7* in single LUAD cells were explored. The results showed that *E2F7* might primarily regulate DNA repair, damage, and cell cycle progression (Spearman coefficients, 0.38, 0.34, and 0.33, respectively; *P *< .001) (Fig. 7A). Based on the data of Kim et al^[23]^ (no. cells = 126), *E2F7* gene expression and the regulatory potential of the 3 aforementioned biological processes were significantly and positively correlated in LUAD (Fig. 7B and C).

## 4. Discussion

LUAD is the most common histological subtype of lung cancer, with a poor clinical prognosis and a high risk of recurrence.^[2]^ Therefore, there is an urgent need to identify novel therapeutic targets and potential prognostic biomarkers for patients with LUAD. Over 30 years ago, E2F was identified and named because of its essential activity in the early events of adenovirus replication and its ability to induce host cell proliferation.^[24,25]^ Since the identification of the first E2F family member (E2F1), 7 additional genes in mammals^[3]^ and various more in other clade, including worms,^[26]^ frogs,^[27]^ flies,^[28]^ and plants,^[29,30]^ have been identified. Since the E2F family plays a crucial role in cell division and DNA synthesis, it influences cell fates and oncogenesis,^[31,32]^. Thus, an increasing number of researchers are focusing on understanding the mechanisms of E2Fs in LUAD tumorigenesis, invasion, and metastasis.

Our study reveals that, according to TIMER2.0-based multiple pan-cancer analysis, the transcript levels of E2Fs are overexpressed in multiple cancer tissues, indicating that these genes are extensively involved in the behavior of cancer. Then, the expression of E2Fs was compared in LUAD and normal samples based on the RNA-Seq data in the TCGA database, and E2Fs were found to be differentially expressed. Additionally, in the OncoPrint visual summary of alteration, E2Fs showed high mutation rates in LUAD patients. Zhang et al^[33]^ reported that meiotic nuclear division 1 upregulates *E2F1* transcription by competitively binding to Kruppel-like factor 6 to regulate cell cycle progression, which is related to tumor development and poor clinical prognosis. Feliciano et al^[34]^ reported that the downregulation of *E2F2* proteins occurs during the expression of miR-99a, and the expression of miR-99 induces apoptosis and cell cycle arrest, inhibiting invasion and migration and facilitating the adhesion of LUAD cells. *E2F3* without 3UTR can resist the influence of downregulated LINC00665 on cell proliferation and invasion and plays a crucial role in the progression of non-small cell lung cancer (NSCLC).^[35]^ In the cytoplasm, the combination of *E2F3* with miR-200b participates in metastasis-associated LUAD transcript-1-mediated docetaxel-resistance, promoting LUAD cell invasion and migration.^[36]^ *E2F5* is regulated by miR-128-2, which enhances the functional gain of mutant p53 by inhibiting lung cancer cell apoptosis and enhancing cancer cell resistance to cisplatin doxorubicin and 5- fluorouracil.^[37]^ Meanwhile, *E2F5* silencing effectively reverses circABCB10-induced NSCLC cell proliferation, invasion, and migration.^[38]^ The downregulation of *E2F6* leads to the upregulation of LINC 01436 expression, promoting lung cancer cell proliferation, migration, and invasion, which has been associated with the OS rate in patients with NSCLC.^[39]^ Increasing the inhibition of *E2F7* promotes the proliferation of LUAD cells and induces G1 phase arrest, which is achieved by the circPRKCI-miR-545/589-*E2F7* axis.^[40]^ *E2F7* expression is regulated by circ-AASDH via sponge miR-140-3p, which participates in tumor proliferation, the clinical stage, and poor clinical outcomes in LUAD.^[41]^ *E2F8*, as a transcription inhibitor of *E2F1* and *E2F2* expression, is an inhibitory regulator of the cell cycle that regulates the Rb-E2F-dependent cell cycle, and the overexpression of *E2F8* seems to be related to poor clinical outcomes in LUAD patients.^[42]^ However, their specific role in LUAD require further investigation.

Bioinformatic analyses can identify additional new prognosis-related genes and can be used to establish more accurate prognostic models than conventional clinical parameters. One means of regression analysis, LASSO, can simultaneously select and regularize variables to improve the prediction accuracy and interpretability of statistical models.^[43]^ This method is widely used to optimize poorly correlated high-dimensional data and master prediction values to avoid overfitting.^[16]^ Thus, it can effectively identify the most effective predictive markers and yield prognostic indicators that predict clinical outcomes. To further identify E2Fs that were significantly associated with prognosis in LUAD patients, we constructed a prognostic model using univariate, LASSO, and multivariate Cox regression analyses to predict OS and PFS in LUAD patients and found that *E2F7* was potentially a prognostic biomarker.

As a pro-tumourigenic transcription factor, *E2F7* is frequently dysregulated in numerous malignancies.^[41]^ *E2F7* was reportedly involved in controlling cell proliferation, cell cycle progression, and differentiation as a transcriptional repressor,^[44]^ which might be the process and mechanism of LUAD tumorigenesis, invasion, and metastasis. We identified that *E2F7* might affect LUAD by regulating DNA repair, damage, and cell cycle processes through CancerSEA. Ye et al also reported that *E2F7* is predicted as the target gene of RAD54L, and this regulatory axis promotes the progress of LUAD, cell proliferation, invasion, and migration through the mTORC1 signaling pathway, and induces apoptosis and G1 cell cycle arrest. Wang et al reported that *E2F7* could rescue the inhibitory effects of sh-SNHG6 or miR-26a-5p mimics on cell migration and EMT, critical for LUAD cell metastasis and EMT.^[45]^ Furthermore, the dysregulation of the SNHG6/miR-26a-5p/*E2F7* axis was positively associated with a more advanced stage, larger tumor size, and worse OS in LUAD patients.^[45]^ Similarly, by univariate and multivariate Cox regression analyses, GEPIA database and immunohistochemical analysis, we also observed that *E2F7* was positively associated with larger tumors and higher TNM stages. This study further confirmed *E2F7* plays an important role in LUAD by multiple analyses, but its exact role in LUAD deserves further investigation.

We are also aware of the shortcomings and limitations of this study. External validation of the clinical dataset would be beneficial, and the biological mechanisms of *E2F7* have not been fully elucidated. Thus, clinical trials and data will be needed for further validation. Additionally, we will attempt to conduct additional external experiments to explore the role of *E2F7* in LUAD in our forthcoming work.

## 5. Conclusion

In conclusion, we systematically demonstrated the correlation of *E2F7* with LUAD by variety of methods, which allowed us to develop a more thorough understanding of the heterogeneity and complexity of the molecular characteristics of *E2F7* in the development and progression of LUAD.

## Author contributions

**Conceptualization:** Shengcheng Lin, Xiangyang Yu, Haojie Yan, Yafei Xu.

**Funding acquisition:** Shengcheng Lin, Xiangyang Yu.

**Investigation:** Zhentao Yu, Shengcheng Lin, Xiangyang Yu, Haojie Yan, Yafei Xu, Kai Ma.

**Supervision:** Shengcheng Lin.

**Visualization:** Zhentao Yu, Shengcheng Lin, Xiangyang Yu, Kai Ma.

**Writing – original draft:** Shengcheng Lin, Xiangyang Yu.

**Writing – review & editing:** Zhentao Yu, Shengcheng Lin, Xiangyang Yu, Haojie Yan, Yafei Xu, Xiaoliang Wang, Yeqing Liu, Ahuan Xie, Kai Ma.

## Supplementary Material
















